# Recurrent Empyema Thoracic Secondary to Pulmonary Nocardiosis in Immunocompetent Patients

**DOI:** 10.1155/2020/8840920

**Published:** 2020-10-26

**Authors:** Samshol Sukahri, Lily Diana Zainudin, Mohd Firdaus Hadi, Mohd Al-Baqlish Mohd Firdaus, Muhammad Imran Abdul Hafidz

**Affiliations:** ^1^General Medical Unit, Department of Medicine, University Technology MARA, Malaysia; ^2^Division of Cardiology, Department of Medicine, University Malaya Medical Centre, Kuala Lumpur, Malaysia

## Abstract

Pulmonary nocardiosis is a rare disorder that mainly affects immune-compromised patients. We report a 37-year-old male who presented with persistent fever associated with productive cough. During this course of therapy, he had recurrent admissions for empyema thoracic. Clinically, his vital signs were normal. Blood investigations show leukocytosis with a significantly raised erythrocyte sedimentation rate (ESR) and C-reactive protein (CRP). Sputum acid-fast bacilli (AFB) was scanty 1+ and sputum mycobacterium culture was negative. Chest X-ray (CXR) showed consolidative changes with mild to moderate pleural effusion on the right side. Skin biopsy was taken and showed Paecilomyces species. A computed tomography scan (CT thorax) was performed and revealed a multiloculated collection within the right hemithorax with a split pleura sign. Decortications were performed and tissue culture and sensitivity (C+S) growth of *Nocardia* species. And it is sensitive to sulfamethoxazole-trimethoprim and completed treatment for 4 months. This case highlights that pulmonary nocardiosis should be kept in mind in also immune-competent patients, especially in suspected cases of tuberculosis not responding to antitubercular therapy.

## 1. Introduction

*Nocardia species* are the bacteria species that can be present anywhere such as in the soil, in the long-standing dust, stagnant water, and the sand. The disease of pulmonary nocardiasis is rarely seen. It was reported that there is an approximate incidence of 500 to 1000 cases annually in the US [1]. The *Nocardia* is a Gram-positive filamentous bacteria and in a group of actinomycetes that can cause opportunistic infection in immune-compromised patients [2]. Nonetheless, it can also infect the immune-competent patients. And an incidence of about 15% has been reported in the US [3]. The infection can be classified as acute, subacute, and chronic infection. Commonly, the infection is acquired through inhalation and could manifest as pulmonary, central nervous system, and cutaneous infections [2, 4]. For the lung manifestation, *Nocardia* may cause a variety of problems such as pulmonary nodules, pulmonary infiltrates, cavitation, and pleural effusion [2]. We reported a case of nocardiosis in a patient presented with recurrent empyema with nonresolving pneumonia.

## 2. Case Report

A 37-year-old middle-age gentleman, who was otherwise fit and well, presented with persistent high-grade fever for more than ten days duration associated with intermittent productive cough of whitish sputum. He also had arthralgia and myalgia. He denied any hemoptysis, night sweats, shortness of breath, or pleuritic chest pain. He did not have any constitutional symptoms such as loss of weight or appetite. He was previously diagnosed with smear-positive pulmonary tuberculosis (PTB) where he completed a total duration of 9 months of anti-TB therapy. During this course of therapy, he had recurrent admissions for empyema thoracic, where a chest tube drainage and prolonged antibiotic therapy were needed. He was a nonsmoker and was doing a rural job, in the form of farming for a living. He denied alcohol consumption and the usage of recreational drugs.

On examination, he appeared tachypneic with a respiratory rate of 24 breaths/minute. His other vital signs were normal. There were reduced air entry and vocal resonance with the presence of stony dullness on examination on the lower zone of the right lung. There was a skin lesion over the face and scalp which resembles seborrhoeic dermatitis. Other systemic examinations were unremarkable.

**Figure 1:**
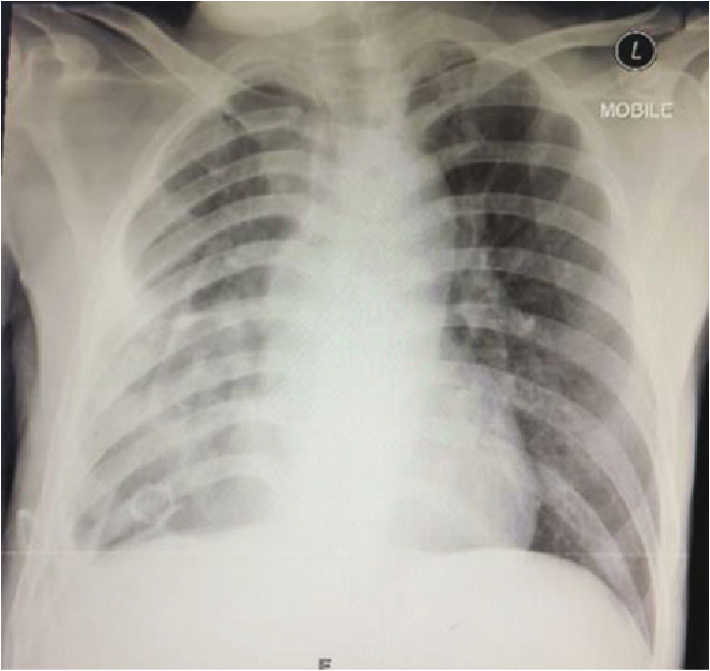
Chest radiography showed consolidative changes with mild to moderate pleural effusion on the right side.

**Figure 2:**
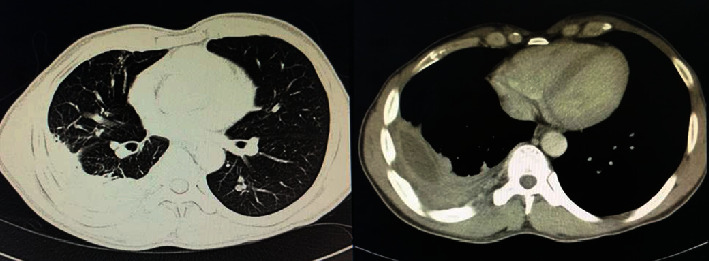
Contrasted enhanced CT thorax revealed a multiloculated collection within the right hemithorax depicting the thickened enhancing wall which joins at the margins of the collection to form the split pleura sign. This is associated with the compression atelectasis surrounding it.

In terms of blood investigations, he had leucocytosis (WCC of 14.9 × 10^9^/*L*) and thrombocytosis (platelet count of 609 × 10^9^/*L*), with significantly raised inflammatory markers (ESR of 102 and CRP of 137.4). A sputum acid-fast bacillus was scanty 1+, and the sputum mycobacterium culture was negative. Hepatitis and HIV screenings were negative. Chest radiography showed consolidative changes with mild to moderate pleural effusion on the right side (Figure 1). The pleural analysis shows the exudative picture with protein ratio (pleural protein) : (serum protein) = (3.3 g/L : 77 g/L) and lactate dehydrogenase (LDH) ratio (pleural LDH) : (serum LDH) = (3317 U/L : 315 U/L). Cytology was sent and no malignancy detected, and an acid-fast bacillus was also negative. Skin biopsy was taken and showed *Paecilomyces species*. CT thorax was performed and revealed a multiloculated collection within the right hemithorax depicting a thickened enhancing wall which joins at the margins of the collection to form the split pleura sign. This is associated with the compression atelectasis surrounding it (Figure 2).

**Figure 3:**
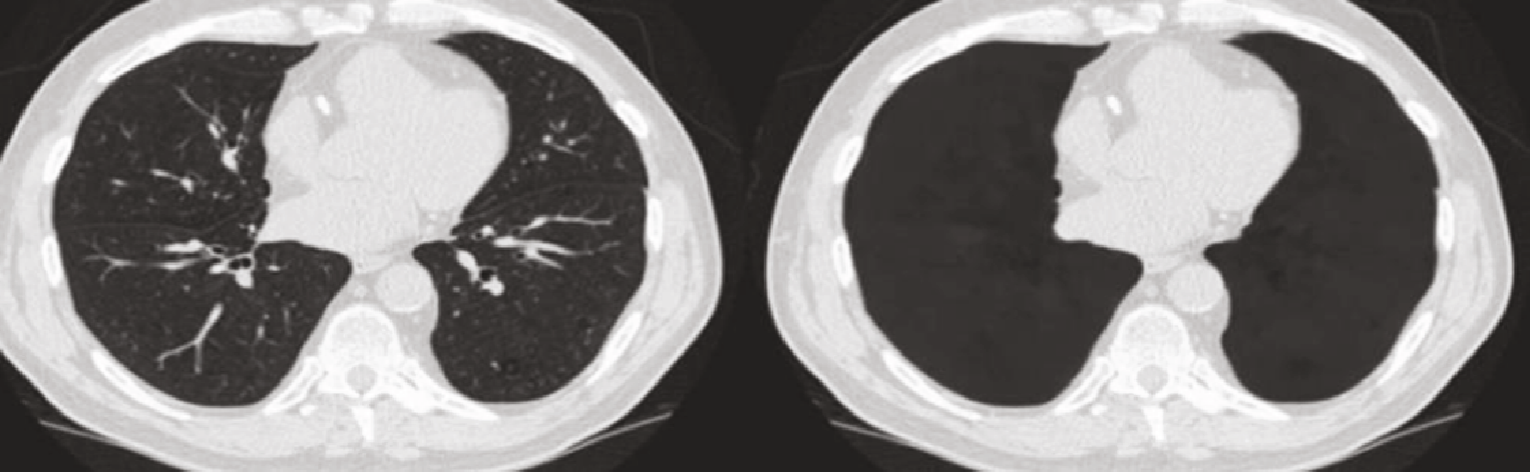
Contrasted enhanced CT thorax postintervention noted resolution of pleural effusion and empyema.

The patient was referred to a cardiothoracic surgeon, and right thoracotomy and decortications were done in February 2016. The findings from the operation are thickened cortex measuring 0.8 cm plastered to the surface of the right lung with small pus collection. Tissue culture and sensitivity were sent and reported as Gram-positive and thin branching filaments. Modified Ziehl-Neelsen staining reported as many branching of acid-fast bacilli which is consistent with the morphology of *Nocardia* species. And it is sensitive to sulfamethoxazole-trimethoprim (Bactrim). He was started on IV imipenem 500 mg QID and oral Bactrim 4/4/3 tablets TDS total for 4 months. After completed treatment, the patient was getting better and no more shortness of breath and cough. Repeated CT thorax noted no more pleural effusion or empyema (Figure 3).

## 3. Discussion

Pulmonary nocardiosis is an uncommon disease that is seen in immune-compromised patients. Dawar et al. reported that the prevalence of *Nocardia* infection in India is 37.5% [5]. A few recognized risk factors had been discovered such as steroid therapy, chronic obstructive pulmonary disease, bronchiectasis, and cystic fibrosis. *Nocardia* also can affect immune-competent hosts. And the reason is because of the impairment of bronchial defences and destruction of its architectures [6]. Both Chronic Obstructive Pulmonary Disease (COPD) and bronchiectasis have been reported as significant risk factors for the colonising of *Nocardia* in the respiratory system [7, 8]. Another possibility is genetic susceptibility. A human gene influences susceptibility or resistance toward the *Nocardia* infection. There is a complex interaction between human factors and environmental factors that regulates immunity toward the infection. The study by Casanova et al. shows that there is a concordance rate of infections between monozygotic and dizygotic twins that compromise the disease susceptibility and the host's genetic background [9, 10]. A retrospective study done by Singh et al. reported that pulmonary nocardiosis found in 52.8% in patients on long-term steroid usage, 52.8% in chronic lung disease, 27.8% in diabetic patients, and 22.2% in solid-organ transplant patients [11]. In our case, the patient was not immune-compromised as he was not on any corticosteroid therapy and not known to have COPD or bronchiectasis. The mechanism of infection, in this case, is fully unknown.

Pulmonary nocardiosis will present a variety of symptoms and nonspecific clinical courses [12]. Hence, fever, shortness of breath, and productive cough are common presenting symptoms. It can also manifest as a skin lesion, but the primary cutaneous nocardiasis is very rare [13]. There are three clinical manifestations: a superficial acute skin and soft tissue infection, a lymphocutaneous infection, and lastly mycetoma. The most common manifestation is mycetoma. As in our case, this patient had a skin lesion that resembles seborrhoeic dermatitis, and skin biopsy revealed *Paecilomyces species*. This species is a saprophytic fungus that can be found in soil and reacts as biodegrading agents. They rarely cause disease to the human unless in immune-compromised patients [14]. This is a possible mode of transmission of pulmonary nocardiosis in this case as he is a gardener and had a lot of works involving soil and plantation. The culture from the skin biopsy is difficult because *Nocardia* is unlike other Gram-positive bacteria; it is a filamentous bacterium with hyphae-like branching on direct microscopy. So it can mislead to fungal infection as in this case [15].

The literature by Frazier et al. shows that nine from twenty positive culture patients of *N. asteroides* have normal chest radiography [16]. A few literatures reported that the commonest chest radiograph findings of pulmonary nocardiasis are fluffy infiltrates; irregular densities; pleural empyema; single or scattered regular or irregular nodules or masses with cavities; single or multiple abscesses; and interstitial, reticulonodular, alveolar, or miliary infiltrates [17–19].

Sato et al. in their study of HRCT in pneumonia patients noted a nodule or mass with interlobular septal thickening and/or cavitation which is suggestive of pulmonary nocardiosis [20]. Other imaging such as CT scan could be performed, and the commonest finding includes consolidation with or without cavitation, multiple discrete pulmonary nodules, pleural effusion, or chest wall extension [21, 22].

The diagnosis of *Nocardia* is difficult because of its slowness of culture growth and along with the lack of a serologic test for nocardiosis. It can mimic pulmonary tuberculosis in both clinical symptoms and radiological characteristics [13]. There is a case report of pulmonary nocardiosis that resembled tuberculosis, in a 35-year-old patient without a definable predisposing condition by Yasar et al. [23]. There could be a false positive result for acid-fast bacilli (AFB) smear as the sensitivity is 68% and the specificity is 90% in the high-prevalence country [24]. As in this case, he was started with anti-TB treatment given his symptoms, chest X-ray, and sputum AFB smear which was scanty positive (1+).

If sputum examinations do not yield the diagnosis in a suspected case and the diagnosis cannot be made easily from lesions elsewhere in the body, more invasive diagnostic procedures like bronchoscopy, needle aspiration, and open lung biopsy should be performed [22]. As in this case, *Nocardia* sp. were revealed after tissue culture was sent from decortications of empyema.

According to the American Association for Thoracic Surgery (AATS) Guidelines for Management of Empyema 2015, patients with chronic empyema need to be managed with decortications (class IIa evidence: decortications are reasonable in patients with chronic empyemas who are medically operable to tolerate major thoracic surgery) [25]. And the British Thoracic Society (BTS) Pleural Disease Guideline 2010 says that patients with persistent sepsis should receive surgical treatment if they have persisting sepsis in association with a persistent pleural collection, despite chest tube drainage and antibiotics [26]. So, prompt referral to specialized unit needs to be done after several admissions for the same problem so that further evaluation and investigation can be done for correct diagnosis and management.

Sulfonamides have been the mainstay of therapy for nocardiosis since the 1940s; trimethoprim-sulfamethoxazole is currently preferred in a dose of 15 mg/kg/day of trimethoprim and 75 mg/kg/day of sulfamethoxazole, either parenterally or orally. Treatment of pulmonary nocardiosis should be continued for 6 to 12 months. Central nervous system disease requires treatment for 1 year unless all apparent disease has been excised, in which case 6 months is sufficient. For immune-compromised patients with nocardiosis, therapy should be continued for 12 months [27]. This case highlights that pulmonary nocardiosis should be kept in mind in also immune-competent patients, especially in suspected cases of tuberculosis not responding to antitubercular therapy and showing no tubercle bacilli either in the direct smears or cultures.

## 4. Conclusions

Pulmonary nocardiosis is a rare infection, especially in immune-competent patients. Diagnosis of *Nocardia* is challenging because of its slowness of culture growth and lack of serologic testing. It can mimic pulmonary tuberculosis; therefore, the result could be a false positive for acid-fast bacilli. We also need to consider *Nocardia* infection if the patient presents with recurrent empyema and not responding to antitubercular therapy.

## Data Availability

The (data type) data used to support the findings of this study are available from the corresponding author upon request.
